# Yes! To Scaling Up Cervical Cancer Screening With Self-Collection: But the Cost of HPV Screening Must Be Reduced

**DOI:** 10.1200/GO.21.00161

**Published:** 2021-08-26

**Authors:** Ann Marie Beddoe

**Affiliations:** ^1^ Icahn School of Medicine, Mount Sinai Medical Center, New York, NY

In 2018, the United States Preventive Services Task Force issued a final recommendation for primary screening of cervical cancer using human papillomavirus (HPV) testing either alone or in combination with cytologic screening in women older than age 30 years. Since then, several agencies including the WHO and the American Cancer Society have echoed the use of HPV testing as the preferred primary screening method beginning at age 25 years, with cotesting or cytology testing alone recommended only if primary HPV testing was unavailable. Currently, only two tests, Cobas (Roche, Basel, Switzerland) and Onclarity (Becton Dickerson-BD, Franklin Lakes, NJ), have received US Food and Drug Administration approval as primary HPV tests and five others as cotests, including Digene's Hybrid Capture II assay (hc2).^1^

Unlike cytology-based screening, molecular detection of HPV collected from cervicovaginal swabs is less influenced by targeted sampling from the transformation zone of the cervix or by specimen adequacy and cell morphology. This has opened the possibility for patients to collect their own HPV samples without undergoing an examination that many perceive as embarrassing. The ability to collect one's own specimens lends a form of empowerment and participation in one's health that is difficult to achieve when asked to undress and be examined in an unfamiliar environment. There is growing evidence that self-collection has benefits that include anxiety reduction, less pain and discomfort, and lower-cost screening opportunities whether implemented through door-to-door kit distributions or in a private room at a health facility.^1,2^

In underserved and hard-to-reach populations, self-collection additionally has been posited to increase access to screening with the potential to decrease social inequities that often pose a barrier to cervical cancer screening.

A meta-analysis by Arbyn et al^3^ demonstrated that sensitivity for self-collected HPV was similar to provider-collected HPV detection when analyzed with polymerase chain reaction but demonstrated lower sensitivity for cervical intraepithelial neoplasia (CIN) 2 with hybrid capture 2 technology on which *care*HPV is based. This has led to questioning whether using *care*HPV for cervical cancer screening with self-collected specimens is the appropriate approach.^2^ Given the additional infrastructure and costs for polymerase chain reaction, *care*HPV is a more cost-effective platform for use in countries with low resources. There is however limited data comparing self- versus provider-collected HPV detection using *care*HPV. Head-to-head comparison using paired sampling of provider- and self-collected specimens of Katanga et al^4^ is therefore important in demonstrating good concordance despite lower positivity rates in self-collected (14%) versus provider-collected (19%) specimens.

Compared with visual inspection with acetic acid, the most common and cost-effective method of screening in low- and middle-income countries (LMICs), sensitivity of vaginal *care*HPV is superior (69.6% and 71.3% for detecting CIN 2 and 3 lesions with *care*HPV versus 21%-73.6% for CIN2+ lesions with VIA). To increase sensitivity of the test, a cutoff of 1.0 has been recommended for vaginal *care*HPV detection, to improve its sensitivity to levels reached with provider-collected specimens.^5,6^ *care*HPV has been widely used in cervical cancer screening programs in developing countries and is an accepted platform providing access to screening in hard-to-reach and rural populations. Results can be obtained within 4 hours, but for cost efficiency, runs must be performed in batches of 90 wells often delaying results until at least the following day.

Although *care*HPV self-collection has high acceptability, and scalability, it comes with some challenges that must be overcome if it is to live up to its WHO prequalified status as an in vitro diagnostics for use in LMICs. There is a steep learning curve for technicians who have never used similar equipment, and on a personal level, two refresher visits by company representative were needed for technicians to feel secure running batches successfully with a minimal number of discarded wells.

The desktop unit is inexpensive and sturdy but given the cost of the reagents and kits, which must be continuously replenished from one single vendor on the African continent, the downstream cost to patients runs between $5 and $7 US dollars per person, which is prohibitive for most women in LMICs. To further scale up HPV screening for cervical cancer in unscreened populations and fulfill the initial target of the 90/70/90 strategy to eliminate cervical cancer, *care*HPV costs must be reduced. It is incumbent upon companies that develop these platforms to ensure that the cost of these tests is more affordable, $2-$3 US dollars, so that women in the most remote villages of the world can have access to HPV screening.
